# Experimental Evidence on Iterated Reasoning in Games

**DOI:** 10.1371/journal.pone.0136524

**Published:** 2015-08-27

**Authors:** Sascha Grehl, Andreas Tutić

**Affiliations:** Institute of Sociology, Leipzig University, Leipzig, Germany; University of the Basque Country, SPAIN

## Abstract

We present experimental evidence on two forms of iterated reasoning in games, i.e. backward induction and interactive knowledge. Besides reliable estimates of the cognitive skills of the subjects, our design allows us to disentangle two possible explanations for the observed limits in performed iterated reasoning: Restrictions in subjects’ cognitive abilities and their beliefs concerning the rationality of co-players. In comparison to previous literature, our estimates regarding subjects’ skills in iterated reasoning are quite pessimistic. Also, we find that beliefs concerning the rationality of co-players are completely irrelevant in explaining the observed limited amount of iterated reasoning in the dirty faces game. In addition, it is demonstrated that skills in backward induction are a solid predictor for skills in iterated knowledge, which points to some generalized ability of the subjects in iterated reasoning.

## 1 Introduction

Recently the question of how humans actually reason in game-theoretical problems has received some attention in the literature [1–3]. Experimental evidence as well as casual introspection suggest that orthodox decision and game theory needs fundamental modifications to bolster its explanative and predictive potential regarding human behavior. In recent years scholars have worked on both providing new models of (interactive) decision making [4–6] as well as identifying the main properties of standard theory which undermine its explanative power [7–9].

Our paper contributes to the second, empirical branch of this literature. Our research focuses on subjects’ ability to perform iterated reasoning, their belief about how well others might do this, and how the subjects are influenced by this belief. We concentrate on iterated reasoning, because many game-theoretical solution concepts such as iterated dominance or backward induction explicitly require that subjects perform at least several steps of iterated reasoning. That is, iteration is directly involved in the definitions of these solution concepts. In addition, even seemingly innocuous solution concepts, such as the Nash equilibrium, in fact involve iterated reasoning in the form of ‘common knowledge’ regarding various aspects of the game, as revealed by epistemic game theory [10]. Hence, iterated reasoning plays a major role in noncooperative game theory, and the assessment of whether humans are actually capable of engaging in iterated reasoning is of great interest.

We are of course not the first to study iterated reasoning in games (see next section). However, our experimental study improves upon the existing literature in the following key aspects. First, previous studies often suffer from the problem that the ability to engage in iterated reasoning and the belief in the ability of one’s co-players cannot be separated properly. That is, if some particular subject shows a limited amount of iterated reasoning, then it is impossible to tell whether this limitedness is due to a bounded ability of the subject or her beliefs regarding the behavior of her co-players. Second, we measure two different forms of iterated reasoning, i.e. backward induction and interactive knowledge, and hence explore whether there is an underlying propensity of subjects to engage in iterated reasoning or if its practice is more specific to concrete decision problems. Third, for each form of iterated reasoning under consideration we obtain multiple observations from each subject while for the most part excluding learning effects. Hence, our measure of the depth of iterated reasoning is in a sense more reliable than typically found in the literature.

The rest of the paper is organized as follows. Section 2 provides a small review of the existing experimental literature on iterated reasoning. Section 3 provides the details on our experimental design. In section 4 we present our results, the fifth section concludes.

## 2 Related literature

Stahl and Wilson [11, 12] as well as Nagel [13] pioneered the systematic study of varying degrees of rationality via experiments. They formulated the so-called level-*k* model which rests upon the idea that players have varying depths of iterated reasoning. In this model, each subject is characterized by a certain level *k* ∈ ℕ, which denotes how many steps of iterated best responses the subjects apply to their belief of what a level-0 player would do. A level-0 type (*L*0) is defined as non-strategic, which means that she has no particular belief about the strategies of others and therefore follows a salient decision rule. A level-1 type (*L*1) believes that all other players are *L*0 types and hence best-responds to this *L*0 strategy. In general a level-*k* type (*Lk*), for any *k* ≥ 1, best-responds to the belief that all others are at maximum level-(*k* − 1) types. Depending on assumptions with respect to the player’s expectations regarding the distribution of her co-players’ types [14, 15] and specification of the *L*0 strategy [16, 17], data on observed behavior in games can be utilized to assign subjects to types (i.e. levels). Generally speaking, in experimental studies using the level-*k* approach levels greater than 3 are rarely observed [18]. Clearly, the level *k*-approach involves iterated reasoning, because higher-level players need to ‘calculate’ all the choices of lower-level players to determine their own choice.

We now turn to the experimental literature that more explicitly refers to iterated reasoning. Two main threads can be identified here. The first one focuses on how many steps of iterated reasoning are performed in general by humans, while the second one pays attention to the process in which subjects can learn to engage in iterated reasoning [19]. Put briefly, it is–as shown by the level-*k* literature–observed that subjects seldom use more than 3 steps of iterated reasoning. With respect to learning, these studies find that both repetition [2, 20] as well as time for reflection [21] considerably improve subjects’ performance on iterated reasoning. Recently, in both strands the influence of general cognitive skills, such as short-term memory capacity or IQ, has also moved increasingly into focus. It turns out that higher cognitive skills positively affect the iterated reasoning performance [1, 22, 23], whereas shocking these skills decreases the performance [23].

Finally, we mention a number other contributions to the literature which relate to our experiment but do not necessarily belong to some identifiable strand. Grosskopf and Nagel [8], Agranov et al. [24], and Carpenter et al. [23] study the question of whether subjects are actually able to find best responses in situations in which the beliefs are either irrelevant (i.e. a weakly dominant strategy exists), exogenously imposed, or measured ex post. Somewhat depressingly, it turns out that many subjects fail to give best responses, even in not-too-demanding decision situations (e.g. two-person beauty contests). Rubinstein [25, 26] advocates the study of response times; he shows over a wide variety of games and decision situations that patterns in response times might plausibly be related to different types of decision procedures and heuristics.

## 3 Experimental design and methods

This section contains all information on the methodological aspects of our study. First, we motivate our experiment against the background of the reviewed literature. Second, we describe our measurement instruments, i.e. the cognitive reflection test, the hit game, and the dirty faces game. Third, we provide our experimental design and outline the course of a session.

### 3.1 Motivation

A common problem in the level-*k* approach as well as in the literature on iterated reasoning is the fact that experimental designs do not allow any differentiation between two possible explanations for the observed limited amount of iterated reasoning [27]. That is, limits in the performed depth of iterated reasoning can be explained by limited cognitive abilities or by beliefs regarding the amount of iterated reasoning performed by the co-players. Note that the level-*k* approach does not necessarily commit itself with respect to the question of whether the types refer to cognitive abilities regarding the depths of iterated reasoning or to the beliefs regarding the behavior of the co-players. An observed level-*k* player might actually be capable of determining many higher levels of best responses, but abstains from doing so because she believes that her co-players are rather unsophisticated. With respect to the literature on iterated reasoning, a similar problem arises because out-of-equilibrium behavior can always be justified by a lack of rationality or by a lack of belief in the rationality of the co-players, as long as no weakly dominant strategies are involved. Hence, the disentanglement of cognitive ability to and belief in iterated reasoning is a natural next step in the empirical study of human behavior in strategic situations.

Surprisingly, the literature on iterated reasoning has made only little effort to assess whether humans have some kind of general capacity to engage in iterated reasoning or the performance crucially depends on the specific cognitive task. Generally, studies focus on one form of iterated reasoning, i.e. either on iterated dominance (beauty contest), backward induction (centipede game and hit game), or interactive knowledge (dirty faces game). This way it was clearly demonstrated that there is a considerable amount of heterogeneity in the displayed depth of iterated reasoning between subjects. However, the question of whether there is heterogeneity within subjects, i.e. whether a subject’s performance in iterated reasoning depends on the form of reasoning, has not been pursued. Many studies only measure one form of iterated reasoning. Even if several forms of iterated reasoning are involved [1, 23], scholars have not pursued the question of the relationship between them.

Finally, in most studies that are not interested in learning effects, only a very small amount of observations are used to assess the performance of individual subjects in interactive reasoning. However, this practice is prone to produce unreliable measures, since subjects can perform well simply due to chance. Hence, it is important to collect multiple measurements and control for learning effects at the same time.

In our study, we want to deal with all of these concerns. That is, we control for the beliefs of the subjects by either using games in which beliefs do not matter or providing the subjects with an exogenous belief. The latter is achieved by substituting the co-players with an algorithm programmed to play perfectly rationally and communicating this to the subjects. Further, we measure subjects’ performance on two forms of iterated reasoning, i.e. backward induction and interactive knowledge. For this purpose, we use the hit game and the dirty faces game (see section 3.2). Lastly, for the sake of more reliable measurement, subjects had to play these games several times. In contrast to learning studies, we try to handicap learning as much as possible by employing a number of countermeasures (see section 3.3).

### 3.2 Measurement instruments

As is common in the literature on iterated reasoning [1, 22], we tried to elicit some cognitive capabilities of our subjects in addition to their behavior in games. For this purpose we picked the cognitive reflection test (CRT) introduced by Frederick [28]. The CRT consists of three questions (see Table 1), which “are ‘easy’ in the sense that their solution is easily understood when explained, yet reaching the correct answer often requires the suppression of an erroneous answer that springs ‘impulsively’ to mind” [28]. We chose the CRT because it seems to be related to the idea of Kahneman [29] and many other researchers [30] that humans have two systems of thought for solving problems, i.e. a spontaneous and hence barely conscious one as well as a slow but more reflective one, which we find intriguing.

**Table 1:** The cognitive reflection test (CRT).

CRT1	A bat and a ball cost EUR 1.10 in total. The bat costs EUR 1.00 more than the ball. How much does the ball cost?
CRT2	If it takes 5 machines 5 minutes to make 5 widgets, how long would it take 100 machines to make 100 widgets?
CRT3	In a lake, there is a patch of lily pads. Every day, the patch doubles in size. If it takes 48 days for the patch to cover the entire lake, how long would it take for the patch to cover half of the lake?

To measure iterated reasoning in the form of backward induction, we use the hit game [2, 20, 23]. The hit game is defined by a number *m* ∈ ℕ and an interval [*a*, *b*] with 0 < *a* < *b* and *a*, *b* ∈ ℕ. Two players alternately pick an integer from [*a*, *b*]. These numbers are added up. The player who reaches *m* or surpasses it wins the game. Since the hit game is a sequential game with complete and perfect information backward induction can be applied to determine the subgame perfect equilibrium: Depending on the game parameters either the first or the second player can ensure a win by consistently forcing the other player into so-called losing positions while the other player is incapable of influencing this outcome (For more details on the solution, see appendix). The hit game provides a straightforward and easy to interpret measure of iterated reasoning; the depth of reasoning required to solve this game can simply be equated with the number of picks of the winning player on the backward induction path. Interestingly, the level-*k* approach somehow fails in this game. Since no specific salient strategy exist, common practice suggests identifying *L*0 with the uniform distribution on the players’ respective strategy spaces [11]. However, at the start of the game one player is in a position to be able to force a win. This player has a weakly dominant strategy, hence already *L*1-players need to apply backward induction perfectly. As a consequence, concerning the player in the winning position, the level-*k* approach can only discriminate between *L*0- and *L*1-types; this fails to capture the intuition that players might very well be able to solve ‘small’ hit games, but fail in hit games of considerable complexity.

We now turn to the dirty faces game, which is commonly used to measure iterated reasoning in the form of interactive knowledge [19, 31, 32]. Each subject is assigned a type, either X or O. Each player knows the types of all others but not her own. However, it is publicly announced that at least one player is an X-type. Then the game proceeds in turns, with subjects privately choosing one of the three possible announcements ‘I am an X-type’ (X), ‘I am an O-type’ (O), or ‘I don’t know my type’ (U). When everyone has chosen an announcement, these are made public and a new turn begins. The aim of the game is to logically deduce one’s own type and to publicly announce it as quickly as possible. Under the condition of common knowledge of rationality, standard arguments from interactive epistemology suggest the following solution [19]: Suppose there are *k* *X*-types. Everybody announces U in turn 1, …, *k* − 1. In turn *k* all X-types announce X, while O-types continue to announce U. In turn *k* + 1 the O-types announce O (For more details on the solution, see appendix). Finally, we note that the depth of reasoning required to solve the dirty faces game differs between X- and O-types, because the O-types deduce their type on the basis of the observed announcements of X-types. For both types we can simply equate the depths of reasoning involved in solving the game with the number of (subjectively) observed X-types + 1 (Adding 1 simply normalizes our measure. That is, in a dirty faces game with exactly one X-player, our measure gives 1 for this X-player and 2 for each of the O-players).

### 3.3 Subject pool, procedure, and design

The experiment was conducted in the experimental laboratory of Leipzig University from fall 2013 through summer 2014. The experiment was conducted in accordance with the Declaration of Helsinki and all procedures were approved by the Institute of Sociology of the Leipzig University. Participants were recruited via the internet recruitment tool hroot [33]. Information about the duration, the payment, and the confidentiality was provided to participants prior to signing up for the experiments. By voluntarily signing up for the experiments via our website, participants provided written consent to participate in the study. Each participant received a EUR 5.00 show-up-fee and could earn additional money during the course of the experiment. In total 269 subjects attended, earning an average of EUR 14.55 in 17 sessions lasting about 1.5 hours.

To begin with, participants received written instructions with general information about the experiment, the fact that communication was prohibited, payment, anonymity, and time restrictions. We pointed out that for each question and decision there would be a guideline time, which could be exceeded but should not be exceeded constantly. Then participants had the opportunity to ask questions about these general rules.

After that the CRT was conducted. The whole experiment was programmed and conducted with the software z-Tree [34]. Participants had 2 minutes for each question. Participants received no feedback between the questions, but at the end of the test the number of correct answers was displayed; these were rewarded with EUR 0.50 each.

Thereafter the instructions for the hit game were displayed on the screen. Participants encountered a practice round (hit0) followed by 7 distinct rounds (see Table 2), which were rewarded monetarily with EUR 0.50 each if won. Concerning the interval [*a*, *b*] from which the subjects had to pick their numbers, *b* was fixed at 3, while *a* alternated between 1 and 2, so as to reduce learning effects. Further *m* increases over time, raising the complexity of the hit games. The complexity of a game refers to the number of correct decisions necessary to win a certain game, i.e. the depth of iterated reasoning required to solve them. Rather than letting the participants play against each other, they played against an algorithm, which was programmed to play the game rationally, i.e. as long as the algorithm was in the loosing position it adds the smallest number, which minimizes the chance for the subject to stay on the winning path by guessing. Once the subject made a mistake the algorithm stayed on the winning path until it won the game.. This fact was communicated to the subjects in advance. We used algorithms for two reasons: First, this way all subjects could start each round in the same (winning) positions, resulting in more interpretable observations, and second, we wanted to rule out possible effects of other-regarding preferences.

**Table 2:** The hit games implemented.

	hit0	hit1	hit2	hit3	hit4	hit5	hit6	hit7
Starting value (*m*)	6	6	11	11	13	13	18	18
Minimum (*a*)	1	2	1	2	1	2	1	2
Complexity (*C*)	2	2	3	3	4	3	5	4

When all subjects had finished the hit games, we handed out the written instructions for the dirty faces game. Participants then had about 15 minutes to study these, to ask questions, and to complete a quiz concerning the payoff structure and game mechanics. Irrespectively of whether the subject answered a question right or wrong, after each question the correct answer and an explanation were provided. If no further questions were asked, we proceeded with the experiment.

To disentangle the influence of ability to engage in iterated reasoning and the beliefs in the iterated reasoning of the co-players, the subjects played both with human co-players (HU version) and with an algorithm (AI version). To control for learning effects, we implemented two experimental treatments: In the AH treatment, the subjects first played with the algorithm and then with fellow subjects, while the order was reversed in the HA treatment.

At the beginning of the AI version subjects were informed that they were playing with an algorithm, which had been programmed to logically deduce its type correctly from the given information and to assume that the subject will do the same. Since subjects were informed about this, they could rely upon the rationality of their (algorithmic) co-players. The observed performance in the AI version could thus be attributed directly to the ability to engage in this kind of reasoning. To rule out subject confusion [27] we stopped a game in the AI version whenever an illogical announcement from the subject would imply that she would observe the algorithm announcing a type which contradicted its true type. This can happen for example when the subject is the only X-type but irrationally announces U in the first turn. The algorithm would then logically correct but de facto wrongly infer that it must be an X-type. In such cases the game stopped after turn 2.

During each new round subjects were randomly and anonymously paired in groups. We varied the group size in both versions across sessions from four to six, to check whether there were any effects of group size. During a session group size was hold constant, hence 112 subjects played in groups of four, 85 in groups of five, 72 in groups of six. To guarantee better comparability between the games of different group sizes, we restricted all games to the seven possible situations someone in a four-person group could be confronted with. These are the trivial situation where the player observes no X-type plus the constellations where the player observes one to three X-types while she is either an X-type herself or not. Further we balance the constellations for the subjects in such a way that they encounter each problem besides the trivial one at least once in both versions of the dirty faces game. Remember that the complexity of a certain dirty faces game is equal to the number of observed X-types + 1. In the AI version subjects simply played all seven constellations, resulting in 269 observations per constellation. In the HU version subjects played, depending on group size, 7 to 10 games. Here another constellation could be observed if group size exceeded four, that is the situation where an O-type observes four X-types (see Table 3).

**Table 3:** The df games implemented and observations in the AI/HU version.

Player’s type	Complexity (*C*)				
	1	2	3	4	5
X	269/111	269/286	269/378	269/332	-/-
O	-/-	269/409	269/409	269/232	-/76*

*only in HU version with group size of 5 or 6

A round ended for a subject as soon as she announced a type or, alternatively, after the sixth turn (theoretically, 6 turns would suffice to solve any constellation of the dirty faces game used in our study). Each player who correctly announced her type got a lottery ticket, which provided the opportunity to win EUR 0.50. If the subject announced the wrong type, she got nothing. The probability that a ticket would win was 100% minus 5% for each time the receiver of the ticket had chosen U in the respective round. This way we guaranteed that announcing one’s type as soon as it was possible on logical grounds was an attractive strategy for maximizing monetary payoffs. To ensure, further, that guessing was an unattractive strategy, players choosing U until the end of the round got a 60% ticket. Such a ticket was better than guessing in the first or any subsequent turn, assuming a 50% probability of guessing correctly. We use lottery tickets instead of direct payoffs due to the fact that in theory individual risk preferences should be ruled out by this procedure. Further, we opted for an individual rewarding based on the actual type (in contrast to making payment conditional on the hole group determining their type correctly [35]) because otherwise we would loose the strategic uncertainty and hence makes beliefs again completely irrelevant in the HU version. To reduce learning effects, no feedback was given between the rounds.

In the last stage of the experiment, the subjects filled in a short questionnaire. They were asked to specify their gender, age, field of study, and previous knowledge of game theory or logic.

## 4 Results

The sample for the entire study was 269 subjects, leading to 1883 observations for the hit game and the dirty faces games (henceforth, df games) in the AI version, as well as to 2233 observations for the df game in the HU version on the level of individual decision making. Nearly two thirds (63%) of our subjects were women and almost all were students (93%). The mean age was 24 years. We asked subjects about their field of study and if they had ever read a book (14%) or attended a course (25%) on game theory or logic. However, besides gender none of these variables turned out to be related to iterated reasoning in any conceivable way.

### 4.1 Cognitive reflection test

**Fig 1:**
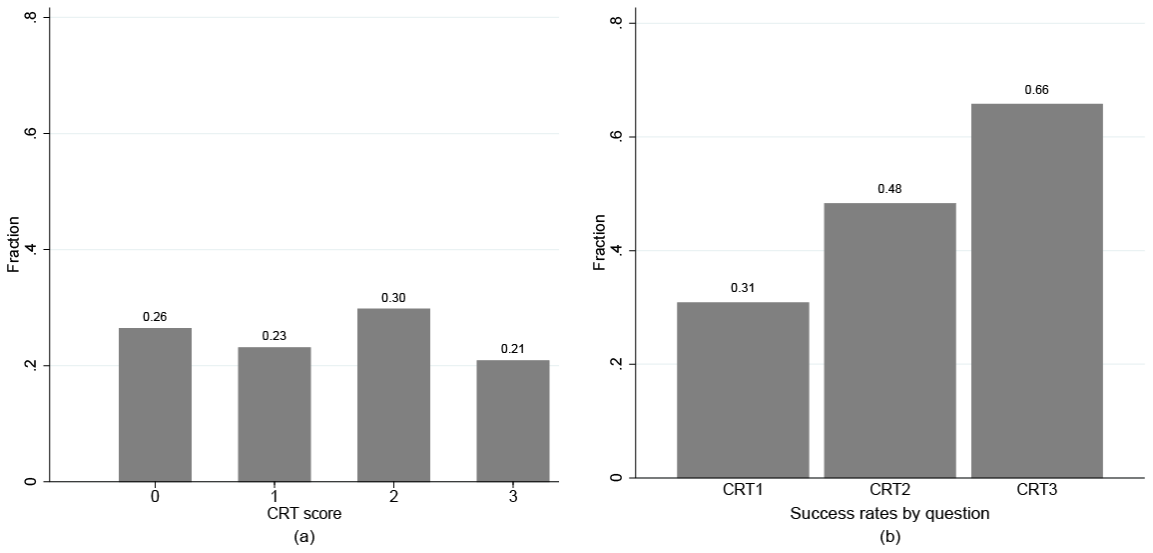
Subjects’ performance in the cognition reflection test.

The numbers of correctly solved CRT questions (CRT score) are relatively evenly distributed, ranging from 21 to 30% (see Fig 1 (a)), which results in a mean score of 1.45 correct answers. Males score on average 0.38 points higher (*p* = 0.005; t-test) and this effect pervades through all three questions. Fig 1 (b) reveals that CRT2 and CRT3 are easier for our subjects. The internal consistency of the construct is acceptable (Cronbach’s *α* = 0.63). Hence, in the following we use the CRT score as a measure for the non-impulsiveness of thinking.

### 4.2 Hit game

**Fig 2:**
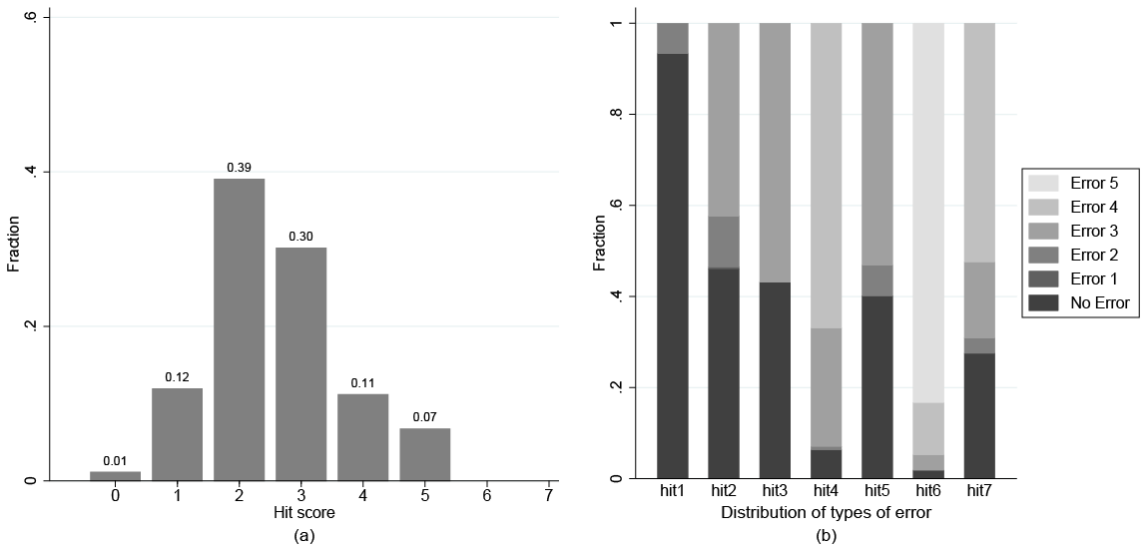
Subjects’ performance in the hit games.

In panel (a) of Fig 2 one can see the distribution of correctly solved hit games (hit score). Over two thirds of our subjects could solve only 2 or 3 problems, resulting in a mean of 2.58 solved games and immediately refuting the idea of perfectly rational actors. Panel (b) provides a compact overview about the observed behavior of our subjects in hit games. To interpret this graph, we introduce the notion of ‘type of error’. An error ℓ refers to a wrong choice in a situation in which ℓ correct choices from the subject are still required to win the game. The graph shows the distribution of errors for each hit game under consideration. For example, consider hit7. To win this game, the subject needs to make four consecutive choices and all of these choices have to be correct. We see that approximately half of subjects fail in the very first decision, i.e. they make an error 4. From the subjects who do not make an error 4, approximately one third make an error of type 3. Then, a small minority of subjects make an error 2. Finally, errors of type 1 are absent in hit7, such that approximately 30% of subjects win the game.

The graph reveals several important patterns in the observed behavior. First of all, error 1 occurs virtually never (only once in hit2). Since an error of type 1 refers to a trivial situation in which the subjects were in a position to win the game directly, this shows that subjects had understood the rules of the game and were motivated to win. More interestingly, the fact that errors of type 2 occurred only rarely means that most subjects are reliably capable of anticipating one action of their co-player. However, the graph also shows that subjects did not substantially outperform random guessing in situations involving the anticipation of two or more actions by the co-player. Consider errors of type 3. Recall that in hit1, hit3, hit5, and hit7 the action space contained only 2 and 3, while in the even hit games the action space contained 1, 2, and 3. Hence, random guessing implies error fractions of 50% for odd hit games and approximately 33% for even hit games. We see that in hit2 and hit7 subjects make less errors of type 3 than predicted by random guessing. In all remaining games, the rate of errors of type 3 roughly equals the rate predicted by random guessing. Hence, there seems to be a small minority of subjects who are able to anticipate two actions of the co-player. Errors of type 4 occur more or less exactly in the proportion predicted by guessing. Surprisingly, errors of type 5 occur more frequently than predicted by random guessing.

A main advantage from our design is that multiple observations on the hit game allow a more reliable measurement of the depths of iterated reasoning in the form of backward induction. To separate success due to guessing from iterated reasoning, we construct an index in which a subject gets the index *i* if she was able to solve at least all games with 1, …, *i* iteration steps involved, but fails at a game with *i* + 1 iteration steps. Table 4 shows the result. Almost every participant could solve problems involving two or less steps of backward induction, but only a very small minority of 6% were able to reliably solve problems involving three steps of iteration. Finally, nobody among our 269 subjects was able to perform four or more steps reliably.

**Table 4:** The *i*-index for the hit games.

*i*-index	Fraction
1	.07
2	.87
3	.06

**Fig 3:**
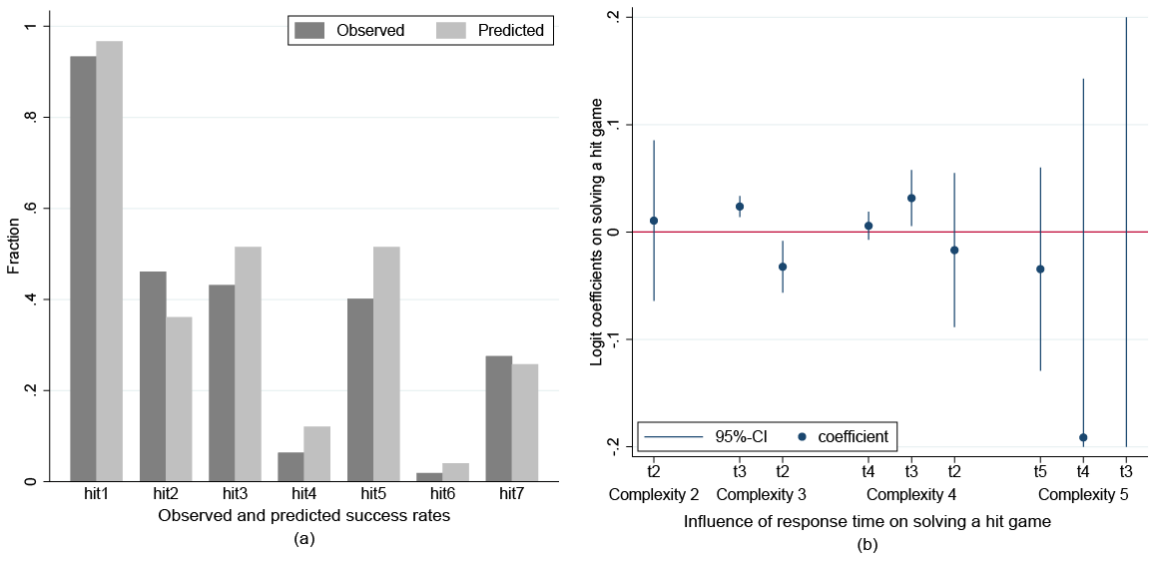
Success rates and response time analysis for the hit games.

Note that our estimates are quite pessimistic in comparison to the literature [13, 36]. We provide two additional sources of evidence to back up our estimates. First, we calculate the expected frequencies of solved hit games based on the estimated distribution of the *i*-index and the assumption that subjects guess randomly in situations that involve more steps of iterations than indexed by their respective *i*-index. The result is shown in panel (a) of Fig 3. Surprisingly, our estimates of the *i*-index combined with the assumption of random guessing in overcomplex situations tend to overestimate the proportion of successful subjects. We conclude that our estimates of the *i*-index do not underestimate the abilities of our subjects for iterated reasoning, but probably overestimate these abilities.

A second source of validation of our estimates comes from an analysis of response times (For the analysis we used pure response times. The analysis was conducted additionally with log response times, but this did not alter the results substantially). Panel (b) in Fig 3 plots the influence of response times on the probability of correctly solving a hit game from logistic regressions which control for gender, CRT score, and size of action space (Fully specified models are provided in Table 5. To achieve a favorable scale the CI is truncated at.2 and -.2, respectively). These regressions were run separately for each decision time grouped by category of complexity. Because each time subjects successfully stayed on the backward induction path the complexity of a game is reduced by one, the different decision times are labeled with a number referring to current complexity of the problem. Note that in each category we dropped the last decision time t1, i.e. the time when a direct win was possible, because, as mentioned earlier, nearly every subject solved this problem, leading to a lack of variance in the dependent variable. In addition, we had to drop t2 for games of complexity 5 for the same reason. Also, note that the estimates of the effects of the response times of decisions are based only on those subjects who did not fail in previous decisions in the respective game.

**Table 5:** Logit regressions on solving a hit game.

	model			
*hit game solved*	(1)	(2)	(3)	(4)
Complexity				
2	2.804**	3.004**	3.016**	3.031**
3 (reference)				
4	−1.267**	−0.948**	−0.947**	−0.957**
5	−3.469**	−2.754**	−2.747**	−2.777**
Size of action space	−0.329**	−0.328*	−0.328*	−0.331*
Response Time				
t2 (sec)		0.019	0.018	0.017
t3 (sec)		0.025**	0.024**	0.023**
t4 (sec)		0.007	0.006	0.005
t5 (sec)		−0.020	−0.022	−0.022
Gender (1 = male)			0.278*	0.211
CRT score				0.198**
Constant	−0.827**	−1.265**	−1.351**	−1.601**
Observations	1883	1883	1883	1883
Pseudo *R* ^2^	0.287	0.299	0.301	0.307

* *p* < 0.05

** *p* < 0.01

The graph reveals that the amount of time subjects take to think about overcomplex problems, i.e. the first decision in games with complexity 4 (t4) as well as the first two decisions in games with complexity 5 (t5, t4), doesn’t matter with respect to the probability of solving the games. That is, these problems are too elaborate for the subjects and hence it doesn’t matter how much time they invest. In stark contrast, the first decision matters for games of complexity 3 (t3), as does the second decision for games of complexity 4 (t3). This conforms to our aforementioned finding that a considerable portion of our subjects are cognitively able to perform the required steps of reasoning in at least some of these games. Additionally, note that decisions in (sub-)games of complexity 2 are not affected by the amount of time invested in making the decisions (In games of complexity 3 we even found a negative influence of response time 2 (t2)). This finding makes sense, because the application of backward induction involves determining each choice right at the start of the (sub-)game which is simple enough to be solved by a considerable portion of the subjects, i.e. (sub-)games of complexity 3. Finally, the fact that the response time of the third decision (t3) in the complexity 5 category does not affect the probability of solving this game, which should be expected according to our reasoning, is most likely due to the small fraction of players who were actually lucky in their first two guesses such that they still had a chance of winning the game (only 14 subjects).

Against this background, we feel that our assessment of our subjects’ skills in iterated reasoning is very solid indeed. We now turn to some other interesting aspects of the observed behavior in hit games. First, note that the hit games used in our study are structurally related in various ways. Some of the simpler games are ‘contained’ in more complex games, which should facilitate the application of backward induction. For example, the reasoning involved in solving hit3 is useful for solving hit5. That is, provided that the subject succeeded in hit3, she knows that the co-player has a winning strategy if she picks 2 in her first choice in hit5. Hence, she knows that it cannot be a bad idea to pick 3, the only alternative to 2 in this game. Similar relationships hold between hit1 and hit3, hit2 and hit4, hit4 and hit6, hit2 and hit6 as well as between hit5 and hit7. We also implemented ‘traps’. Hit2 and hit3 both have *m* = 11, but the minimal pick equals 1 in hit2 and 2 in hit3. Consequently, backward induction dictates picking 3 in hit2 and 2 in hit3 as the respective first decision. The pairs hit4 and hit5 as well as hit6 and hit7 are traps too.

Indeed we find evidence for both backward induction as well as for sloppy, short-cutting reasoning. Table 6 contains the results of *χ* ^2^ based measures of association. First, note that all of the significant associations are descriptively positive. In three instances we find strong associations. The fact that the hit1 and hit3 are not related is not too troubling, considering that there is almost no variance in hit1. More puzzling is the finding that solving hit2 does not facilitate the solutions of hit4 and hit6. We speculate that this is due to our ‘trap’, which might somehow undermine the faith of our subject in the use of their solution of hit2 for more complex hit games. In fact the first trap worked fine; we find a strong negative association between hit2 and hit3, i.e. *χ* ^2^ = 33.606, *p* < 0.001 and *ϕ* = −0.354. The other two traps did not work, most likely because the subjects had learned their lesson.

**Table 6:** Pearson’s chi-squared test for independence.

hit games	*χ* ^2^	*p*	*φ*
hit1—hit3	0.014	.907	−0.007
hit2—hit4	1.183	.277	0.066
hit2—hit6	1.396	.237	−0.072
hit3—hit5	5.606	.018	0.144
hit4—hit6	9.762	.002	0.191
hit5—hit7	18.137	.000	0.260

Table 5 concludes our analysis of the hit game. It contains the results of three logistic regressions which estimate the probability of solving a hit game as a function of parameters of the hit game (complexity and size of action space) as well as individual characteristics of the subjects (gender, CRT score) and response time (Since games from a given subject are likely correlated, we also ran a random effects logistic regression. However, these results did not differ substantially). We find that the complexity of a hit game is the strongest predictor for success in solving it. The effect of the size of the strategy space is a nice indicator of guessing. As already indicated by our previous analysis, response time only matters for problems of complexity 3. Problems of complexity 2 seem to be trivial, i.e., there is no benefit in spending time on thinking about them. Problems of complexity 4 or 5 are overcomplex, i.e., thinking about these problems is just a waste of time. For simplicity, our models only estimate a global effect of non-intuitive thinking (CRT score). We find that non-intuitive thinkers fare better in hit games. However, nonreported analyses show that CRT score matters for simple problems but not for complex problems. As a consequence, the effects of CRT score in Table 5 are quite modest. Note also that intuitive thinkers take less time to make first decisions in hit games (about 10.3 sec. per CRT point, *p* = 0.013; OLS-regression), but this does not exhaust the effects of CRT score. Finally, we observe that males do better in the hit game (i.e. they solve on average 0.38 more hit games, *p* = 0.006; t-test). However, these differences vanish if we take into account that males take more time to think about their first decisions (i.e. on average they take 30 percent more time than females, *p* = 0.001; t-test) and do not rely on intuitions as much as females do (see Sect. 4.1).

### 4.3 The dirty faces game

Recall that our prime interest regarding the df game is to compare the HU version with the AI version. This allows us to separate the effects of cognitive ability to engage in iterated reasoning from the expectation that the co-players engage in iterated reasoning. More specifically, we want to estimate the fraction of observed behavior in df games which can be explained by the ‘theory’ that common knowledge of rationality provides. Of course, for any specific dirty faces game we don’t need common knowledge of rationality. It suffices that a statement of finite length of the form ‘Everybody knows that everybody knows that everybody knows…. that everybody is rational.’ is true. Common knowledge of rationality is needed to guarantee the solution for any dirty faces game. In the AI version, the only part of the theory that can fail is the subject’s rationality, whereas in the HU version common knowledge of rationality might fail additionally because the subjects lack necessary higher order beliefs in the rationality of the co-players. Of course, realistically we expect that beliefs do not fail only on higher levels, but on the most basic level, i.e., we expect that subjects do not believe in the rationality of the co-players. In this sense, the comparison of the rates of behavior which can be explained by common knowledge of rationality (henceforth, ckr-behavior) between the AI and the HU version allows us to estimate the relative importance of ability to engage in iterative reasoning and that of beliefs in the performance of one’s co-players.

However, the fact that errors of co-players occur in the HU version but not the AI version requires some attention. The basic question is whether an error of a co-player is observable for a player or not. If an error occurred but is not observable by the player, she can still act rationally on the belief that her co-players are rational, and on subsequent higher order beliefs of rationality. Hence, her behavior can still be reasonably judged against the standards of ckr-behavior. Note that ckr-behavior under conditions of unobservable errors of co-players might involve announcing a type which contradicts her factual type. To see this, consider a two-person df game with one X-player and one O-player. If the X-player erroneously announces U on the first turn, an O-player believing in the rationality of her co-player should announce X on the second turn. In the following, the notion of individually correctly solved df games does not refer to factual correctness, but to ckr-behavior in the sense described (Alternatively, we could drop these observations from our data set. However, since unobservable errors occur frequently, we would lose a considerable amount of our data).

Turning to observable errors, two types of errors have to be distinguished, i.e. errors by X-players and errors by O-players. Preliminary analysis of our data showed that cases with observed errors of O-players are very similar to cases without observable errors in terms of the estimated fraction of ckr-behavior. Hence, we include these cases in our analysis. Matters are different for errors by X-players. In most of these cases, theory does not provide a satisfactory solution in terms of ckr-behavior, which is why we exclude them from our analysis.

We now turn to the analysis of observed behavior in df games. Subjects correctly solved 36.6 and 41.9% of the AI and HU games respectively, refuting again the idea of perfectly rational actors. In Table 7 we categorize these results by iteration steps required and subject type (Subjects who announced their type before they could logically deduce it (i.e. guessing), cannot be assigned to a type. However, these subjects are included in the ‘total’ columns). We observe the same pattern in both versions: The more iteration steps involved, the lower the fraction of subjects that could solve the puzzle. In addition we see that is was more complicated for subjects to solve a game when they were an X-type. Note that this finding runs counter to theoretical predictions. It is plausible that this effect is due to different degrees of salience regarding the informational value of co-players’ decisions. If a subject observes that all X-types announce X, this is more thought provoking (Why do they know their types already?) than if the X-types signal U (Why don’t they know their types yet?).

**Table 7:** Solved df games by complexity, version, and player’s type. Observations in parenthesis.

Complexity	AI version			HU version		
	by type		total	by type		total
	O	X		O	X	
1		.95	.95		.78	.78
		(269)	(269)		(111)	(111)
2	.57	.44	.49	.83	.65	.70
	(256)	(261)	(538)	(314)	(347)	(691)
3	.32	.11	.21	.46	.14	.22
	(256)	(262)	(538)	(160)	(390)	(575)
4	.20	.05	.12		.08	.07
	(246)	(245)	(538)		(333)	(382)
5					.04	.03
					(27)	(34)

At this point we want to stress that in comparison to our discussion of the observed behavior in the hit game, guessing only plays a minor role in the df game. Due to our design, guessing was less profitable than simply picking U each round. Our payoff structure implies that guessing gets even more unattractive on later turns of a round. Hence, looking at early announcements of types which cannot be justified by logical deduction provides a good estimate for the prevalence of guessing in our data on the df game. We find that about 5% of individual plays of df games involve guessing. Note that, in our descriptive statistics announcements of types before these types can actually be deduced logically are classified as non-ckr-behavior.

For convenience, the situations a subject is confronted with will further be labeled *mT*, where *m* denotes the number of observable X-types and *T* denotes the type of the subject. For simplicity the number of observable O-types is neglected. Interestingly, subjects perform worse in the HU version of the 0X game than in both the AI version of the 0X game as well as in the 1O game. This is striking because it is the only situation where beliefs in the rationality of co-players is completely irrelevant. We suggest that this effect is due to some ‘play instinct’ (i.e. ludic drive) to trick their co-players to erroneously announcing X on the second turn. In fact the trick worked really well, since 71 of 92 co-players in these situations actually announced X on the second turn. Ironically, all but 1 of 24 trickers announced X on the second turn as well. In a nutshell, the trickers traded a five percent chance of winning EUR 0.50 for the pleasure of outwitting their co-players ([35], who raffle 75 Australian dollars per person, observe no single case where the 0X problem has not been solved). Also, subjects did not trick the algorithm perhaps because there is no joy in fooling machines.

**Fig 4:**
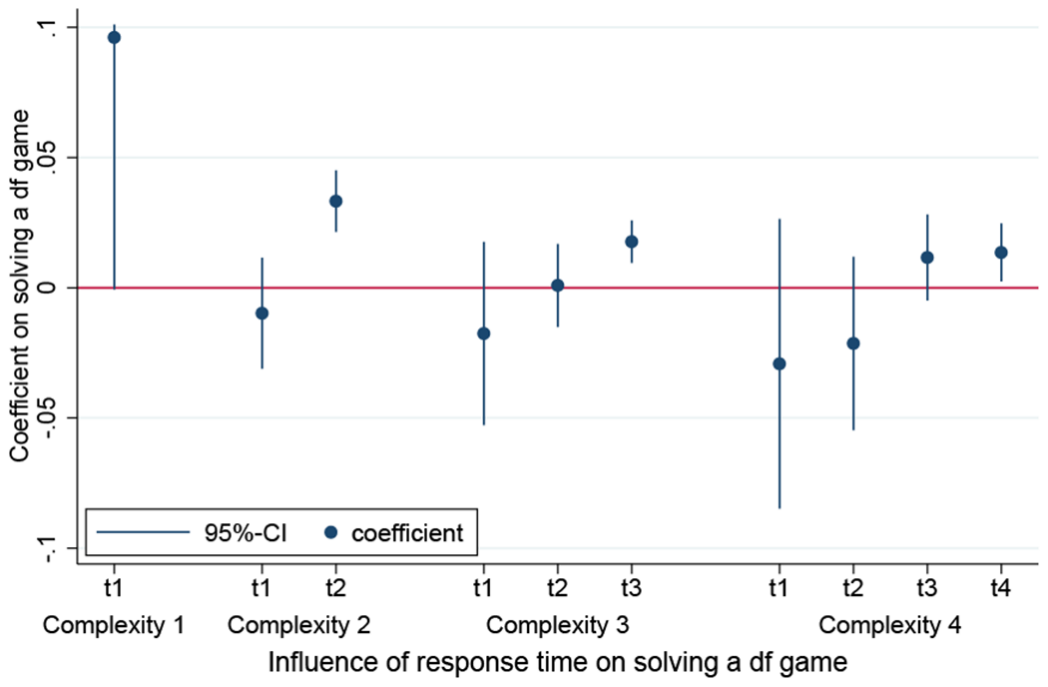
Effect of response time on solving a df game.

Similarly to our analysis of the hit game, we now turn to the influence of response times in the df game. Fig 4 plots the coefficients and 95% confidence intervals of response times as estimated by random effects logistic regression models. In these models, the probability of solving a df game of a specific complexity is regressed on the amounts of time subjects invest on each turn and control variables (We deal with the effects of these control variables in a subsequent paragraph). Note that both AI and HU versions of the df game as well as both treatments are pooled in these regressions. Also, note that the theoretical solution based on common knowledge of rationality involves that a player in a game of complexity *c* (recall, *c* equals the number of observed X-types + 1) announces U in the first *c* − 1 turns and her type on turn *c*. Hence, the graph depicts *c* coefficients and confidence intervals for games of complexity *c*. Finally, we remark that games of complexity 5 only occurred in the HU version, and in only 34 cases no critical error by a co-player destroyed the solvability of the game for the player (see Table 7). Since these cases do not suffice for regression analyses, games of complexity 5 are not included in our graph.

A straightforward conclusion emerges from the analysis of response times. Response time only matters if invested on the crucial turn, i.e. the turn on which it is de facto possible to logically deduce one’s own type. It is worth noting that subjects invest considerable amounts of time on the noncrucial turns (i.e. on average about 16 seconds), but these investments have no payoff. This finding, in a sense, parallels our findings with respect to the hit game. In the hit game, response time only affected games of complexity 3. Regarding the df game, we find that in situations in which the announcements of the co-players have not yet allowed the deduction of one’s own type and hence situations which involve contingent thinking (e.g. ‘If all the X-players announce X in turn 3, I will announce O in turn 4, because […]’), response times have no effect. To us it seems that this means that subjects do not engage or do perform badly in this kind of contingent and hence complex thinking tasks. In contrast to these situations in which only thinking involving contingencies is useful for the solution of the problem, on the crucial turn all the ‘facts’ regarding the types of the other players are on the table. That is, on the crucial turn no contingent thinking is involved the subjects merely have to properly infer their own types from the available information.

It is time to turn to the main research question of our paper. What factor accounts more for the observed limits in performed iterated reasoning: Limited cognitive abilities to engage in iterated reasoning or lack of beliefs in the abilities of the co-players? Table 8 shows the observed frequencies of ckr-behavior for both AI and HU versions of the df game and both treatment conditions. Most importantly, there is no evidence that the beliefs of the subjects regarding the rationality of their co-players is of any importance in the df game. That is, with some occasional exceptions, the frequency of ckr-behavior is not greater in the AI version than in the HU version of the various df games. In fact, for more experienced subjects, i.e. comparing the HU version in the AH treatment with the AI version in the HA treatment, Table 8 even suggests that in the HU version there is more ckr-behavior than in the AI version. However, this might as well be due to learning effects which might depend on the order of versions. That is, it is plausible that subjects have better chances of learning the game while playing with algorithms than while playing with human co-players. For example, 3O situations did not arise in the HU version of the HA treatment (because of errors by co-players) and hence subjects could not gain any experience for these situations in the HA treatment.

**Table 8:** Solved df games by treatment. Observations in parenthesis.

Complexity	AH treatment				HA treatment			
	AI version		HU version		HU version		AI version	
	O	X	O	X	O	X	O	X
1		.95		.77		.81		.95
		(193)		(79)		(32)		(76)
2	.48	.40	.88	.72	.73	.40	.80	.52
	(187)	(188)	(233)	(262)	(91)	(85)	(72)	(73)
3	.32	.11	.49	.17	.00	.09	.35	.11
	(184)	(187)	(152)	(256)	(8)	(134)	(72)	(75)
4	.22	.07		.12		.00	.16	.00
	(173)	(172)		(223)		(110)	(73)	(73)
5				.06		.00		
				(16)		(11)		

Both the unimportance of beliefs in the rationality of the co-players as well as the treatment effects on learning can also be seen from the *i*-index on the df game (Table 9). Interestingly, the *i*-index is generally higher in the HU version than in the AI version. Hence, beliefs in the rationality of the co-players seem to play no role in the df game. Note that the *i*-index of the AI version in the AH treatments roughly equals the *i*-index of the HU version in the HA treatments. So, for inexperienced subjects it does not seem to be too important whether the co-players are algorithms or humans. However, as already indicated, learning seems to be easier if playing with rational co-players. This can be seen from the fact that the *i*-index of the HU version of the AH treatment puts more weight on higher indices than the *i*-index of the AI version of the HA treatment.

**Table 9:** The *i*-index for the df games by treatment and version.

*i*-index	treatment					
	both		AH		HA	
	AI	HU	AI	HU	HU	AI
0	.05	.09	.05	.09	.08	.05
1	.64	.40	.70	.31	.63	.49
2	.25	.43	.19	.50	.25	.38
3	.06	.05	.05	.05	.04	.08
4	.01	.03	.01	.04	.00	.00

Since we have two *i*-indices for each subject on the df game, i.e. one index for both the AI and the HU version, and these indices refer to chronologically ordered behavior, we can check learning on an individual level. That is, we can look at the proportion of subjects who hold constantly of their level, improve their level, or degenerate. In the HA treatment 58% of subjects hold their level, 32% improve, and 10% actually manage to decrease their level. In the AH treatment, 45% of subjects hold their level, 44% improve their level, and 11% decrease their level. Again, this finding suggests that learning is more efficient in the AH treatment, in which the first part of the treatment confronts the players with rational co-players.

To back up our impressions from descriptive statistics regarding the role of beliefs in the rationality of the co-players and the treatment effects of learning, we estimated a family of random effects logistic regressions. The dependent variable in these regressions is the probability of ckr-behavior in the df game. Table 10 shows three regressions; the first deals with structural variables of the df game, the second adds variables related to treatment and procedure. The final model also incorporates individual characteristics of the subjects, in particular CRT score and hit score.

**Table 10:** Random effects logistic regressions on solving a df game.

	model			
*df game solved*	(1)	(2)	(3)	(4)
Complexity	−2.023**	−2.319**	−2.319**	−2.318**
Own type (1 = X)	−1.181**	−1.141**	−1.140**	−1.143**
Group size	−0.062	−0.141	−0.127	−0.116
Round		0.130**	0.130**	0.129**
Version (1 = HU)		−0.060	−0.060	−0.058
Treatment (1 = HA)		−0.426*	−0.389*	−0.410*
Response time (sec)		0.019**	0.019**	0.018**
Gender (1 = male)			0.427**	0.210
CRT score				0.368**
Hit score				0.216*
Constant	3.697**	3.093**	2.859**	1.805**
Observations	3480	3480	3480	3480

* *p* < 0.05

** *p* < 0.01

First of all, structural features of the df game have no surprising effects against the background of our descriptive findings. That is, complexity and being an X-type diminish the probability of ckr-behavior. Notably, the number of co-players does not. This finding already suggests that beliefs about the rationality of the co-players might be empirically irrelevant. The second block of variables teach important lessons. Most notably, it does not matter empirically whether the subjects play with possibly irrational co-players or with rational algorithms. This answers the major motivational question of this paper: Beliefs about the rationality of co-players are irrelevant for the form of iterated reasoning involved in the df game. In addition, we see two kinds of learning effects. On the one hand, experience in the df game benefits ckr-behavior. On the other hand, there is an additional treatment effect, i.e., ckr-behavior is more common when subjects learned the game by playing with algorithms instead of humans first. This interpretation suggests an interaction effect between experience (number of rounds already played) and treatment. Admittedly this interaction effect does not gain significance, which is why it is not reported in these models. Still, we feel that our interpretation is plausible in view of the reported descriptive statistics (see Table 9).

Finally, as already reported, the time subjects invest during the crucial turn benefits ckr-behavior. Model 3 and 4 adds personal characteristics, i.e. gender, CRT and hit score. Similarly to our finding in the hit game, males do better in the df game, although once again these difference can be explained by the fact that males take more time to come to decisions on the crucial turn and faring better in the CRT. More important than the role of gender is the question of whether our experiments provide evidence for some form of generalized cognitive skill in iterated reasoning. And indeed, we find that subjects who performed better in hit games, i.e. iterated reasoning in the form of backward induction, show more affinity to ckr-behavior in the df game. Finally, intuitive thinkers do worse in df games, as they did in hit games.

## 5 Conclusion

This paper provides experimental evidence on iterated reasoning in games. Three main lessons emerge. First, subjects who do better in backward induction also do better in problems involving interactive knowledge. This suggests that there might exist some generalized ability to engage in iterated reasoning. Second, due to the fact that we took multiple measurements of both forms of iterated reasoning under consideration and also controlled for learning effects, we were able to provide quite reliable measurements of subjects’ skill. In comparison to the literature, our estimates of subjects’ skill in the hit game [36] and the df game are rather pessimistic [19]. Third and most importantly, our design sheds light on the question of which factor–cognitive ability or beliefs in the abilities of one’s co-players–is more important to explain the small amounts of iterated reasoning observed in the literature. We find that in the df game beliefs in the rationality of the co-players are completely irrelevant. In addition to these substantive insights, on methodical grounds this paper exemplifies the usefulness of response time analysis to validate estimates of subjects’ abilities in cognitively demanding tasks [25, 26].

Clearly, our main finding regarding the relative importance of cognitive abilities and beliefs in the rationality of co-players cannot be easily generalized to other types of games and subjects. It might well be that the df game is far too complex to allow more or less inexperienced subjects to engage in reasoning about the rationality of their co-players. It is therefore important to use similar designs with more experienced subjects or simpler games [37]. In any case, we feel that more experimental efforts should be directed at assessing whether and to what extent humans actually take into account the perceived weaknesses in the rationality of their co-players, because this question seems vital for an empirically oriented game theory, i.e. a theory of interactive decision making that actually captures how real actors play games.

## Supporting Information

S1 Instructions Instruction materials given to our subjects. Translated from German.
(PDF)

S1 Appendix Extended descriptions of the hit game and the dirty faces game. (PDF)

S1 Dataset Used data in csv format. (CSV)
