# Supplementary material for: Experimental Evidence on Iterated Reasoning in Games
Source: PLoS One. 2015 Aug 27;10(8):e0136524. doi: 10.1371/journal.pone.0136524 (PMC4552469; doi:10.1371/journal.pone.0136524)
Supplement: S1 Instructions — Translated from German. (PDF) [file pone.0136524.s001.pdf]

S1 Instructions

The following are all instruction materials given to our subjects. Note that these instructions have been translated from German. Also, this instruction material refers to the AH treatment.

Introductory instruction (on paper)

Thank you for taking part in this experiment. All of your decisions will be treated anonymously and confidentially. Please note that it is forbidden to communicate with other participants during the entire experiment. Failure to comply with this rule may result in your suspension from the experiment. In addition we ask you to mute or switch off your mobile phones and any other electronic devices which could disturb the process of the experiment.

If you read these instructions carefully, it will later be easier to earn extra money. That will happen if you get extra points because of specific actions during the experiment, for example if you solve a task. For each point, you will get one euro cent at the end of this session. You can see your score in the top left corner of the screen during the experiment. It will be updated only after each part of the experiment.

The experiment is subdivided into 4 parts. These will all be conducted with a computer program. Certain parts of the experiment will be iterated several times. Such an iteration is called a round. The rounds proceed in turns. You have to make one decision on every turn. In some parts of the experiment all participants will decide simultaneously, while in other parts participants decide one after another. You be will be told in due time which procedure applies.

You can see in the top right corner of the screen how much time remains for making a decision. It's no problem if you need a little bit more time. However, to finish the experiment in time, we kindly ask you to exceed the time limit only occasionally.

Keep this instruction sheet next to you during the experiment and take a look at it if you are unsure of the procedure. If you have any questions after reading these instructions, just give us a sign. An assistant will come to you and answer your questions.

At the end of the session, a number will be displayed on your screen. Please memorize this number and come to the staff when you get a signal. Your payoff can only be determined with the aid of this number. Don't forget to hand us back all instruction material you receive during the experiment.

Structure of the experiment:

- Please read these instructions carefully. Call us if you have any questions.
- Part 1 of the experiment
- Part 2 of the experiment
- Instruction paper for parts 3 & 4 of the experiment
- Quiz for parts 3 & 4 of the experiment
- Part 3 of the experiment
- Part 4 of the experiment
- Questionnaire
- You can see your number on the screen. The session is finished and you get your payoff.

Instructions for part 1 (on screen)

In this part of the experiment you have to answer three questions. Each correct answer is worth 50 points. Note that you have two minutes for every question.

Instructions for part 2 (on screen)

In this part of the experiment you and a co-player take turns in removing a certain number of balls from an urn. If you take the last ball in the urn you win 50 points, if this is achieved by your co-player you get no points. The maximum number of balls that can be taken per turn is limited to 3. The minimum number of balls that must be taken per turn is either 1 or 2, depending on the round in question.

Your co-player is a computer algorithm, which is programmed in such a way, that it tries to win; in order to achieve this goal it will plan several steps ahead.

The first round will be a practice round, which does not affect your payoff. Use this round to become acquainted with the program. Thereafter you will play seven rounds, which may differ in the initial number of balls in the urn and in the minimum number of balls that both players must remove

per turn. Again, the maximum number of balls which can be removed will always be 3.

Note: You do of course win even if you have to take more balls than available. This can happen if you have to take at least 2 balls, but only one ball is left.

Instructions for part 3 & 4 (on paper)

Summary

Imagine the following scenario: You and three other participants each flip a coin. Afterwards, you and each of the other three participants may see each other's outcomes of the coin flips, but nobody is allowed to see the outcome of one's own coin flip. After that, all participants have to find out whether their own coin shows heads or tails, on the basis of a public announcement as well as communication with fellow participants via computer terminals.

Details

In each round you have to form a group with 3 randomly selected participants. Afterwards, you and your co-players each flip their coin (both group formation and coin flips are realized via the computer terminals). A coin can show heads or tails. Each side has a probability of 50 %. You are allowed to see the coins of your co-players, but not your own coin. Your co-players are in the same situation: They can see the coins of all other co-players (including your coin), but not their own coin.

After that, there will be a public announcement for you and your co-players. It says either 'at least one coin shows tails' or 'at least one coin shows heads' (Attention: Only one statement will be given, although both might be right).

Your task is to deduce, what you can say about your own coin. Each of the players has to choose between the following statements on every turn

- 'I don't know if my coin shows heads or tails' (below: 'Unknown')
- 'My coin shows heads'
- 'My coin shows tails'

If all players have made a decision, these decisions will be published at the same time. The decisions of your co-players contain new information for you, which you can use on the subsequent turns of the respective round.

As soon as you pick either heads or tails as well as after picking 'Unknown' six turns in a row, the respective round ends for you. You get a

lottery ticket at the end of each round. Every winning ticket has a value of 50 points, however the probability of winning depends on your choices in the respective round. At the end of the final round, it will be revealed which of your tickets win and which lose.

Your last decision in a round determines the winning chances of your ticket for this round:

- ‘Unknown’: You get a ticket with a winning chance of 60 %.
- You picked the wrong side of the coin: You get a ticket with a winning chance of 0 %.
- You picked the right side of the coin: You get a ticket with a winning chance of 100 % minus 5 percentage points for each turn on which you have chosen ‘Unknown’.

It is a fair coin, i.e., both heads and tails appear with equal probability. Because of that, guessing does not payoff for you. If you guess, you have a 50 % chance of winning a lottery ticket with a nonzero winning chance. However, you get a lottery ticket with a chance to win of 60 % if you pick ‘Unknown’ on each turn of the round.

Structure of the computer program

The surface of the program is subdivided into three areas, which are marked by different colors at the left edge of the screen. These areas are:

- An area containing private information (red), which only you know personally. The others can get similar information.
- An area containing public information (blue), which is visible for all of your co-players. For example, this area contains the aforementioned public announcements.
- An area in which you make decisions (green). Here you can make your decision on every turn. In addition, this area includes information about the available lottery tickets.

Figure A shows the private area where you can see the coin flips of all players. The sides of the coins will be determined randomly at the beginning of a round. Figure A exemplifies a specific situation as it might come about in the actual experiment. You can see that the coins of player 2 and 4 show heads (represented by the letter K) and the coin of player 3 shows tails

(represented by the letter Z). But you can't see the side of your own coin (represented by the question mark ?).

Of course each of your co-players also has such an area, but the information displayed differs between the players. So player 2 also sees the coins of players 3 and 4, but he can also see the side of your coin. However, player 2 can't see his own coin.

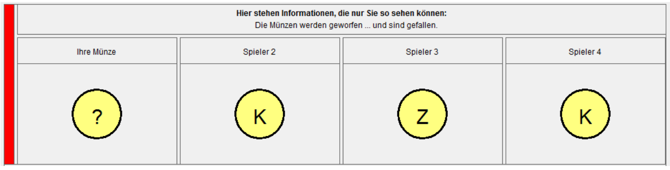

Figure A

Figure B shows the first part of the public area, where you can see the public announcements. In this case it is 'At least one coin shows heads'.

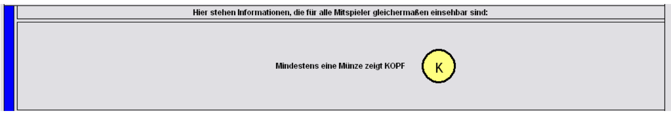

Figure B

Figure C shows the second part of the public area, where you can see the decisions of all players during this round. In this specific situation, on turn 1 all players stated that they don't know whether their coin shows heads or tails respectively (represented by the text 'Unknown'). On the second turn, players 2 and 3 stated that their coin shows heads, while all of the other players picked 'Unknown' again. You can see no information on turns 3, 4, and 5. Of course, this means that the current turn is 3.

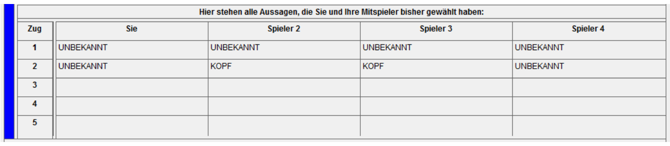
Hier stehen alle Aussagen, die Sie und Ihre Mitspieler bisher gewählt haben:

Zug	Sie	Spieler 2	Spieler 3	Spieler 4
1	UNBEKANNT	UNBEKANNT	UNBEKANNT	UNBEKANNT
2	UNBEKANNT	KOPF	KOPF	UNBEKANNT
3				
4				
5				

Figure C

Figure D shows the area where you can choose one of three possible statements. In addition, there is information about the lottery tickets you would obtain if this round ended for you on the current turn.

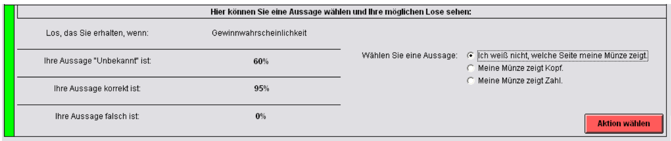

Figure D

Questions

If you have any questions, please feel free to contact us.

Instructions for part 3 (on screen)

This part of the experiment is carried out as described on the instruction sheet. However, some details are different. Therefore, please read the following information carefully:

- The human co-players are replaced by a computer algorithm. The algorithm is programmed to always draw the logically correct conclusion from the available information. Note that the algorithm is not omniscient, but has exactly the same information as a human player would have. The algorithm is programmed to announce the side of its coin as soon as it can logically do so. Furthermore, it assumes that each co-player (including you) also draws logically correct conclusions only.

- Please note that, in this part of the experiment, it may happen that a round ends before reaching the 6th turn, even if you only picked 'Unknown'.

- This part of the experiment consists of 7 rounds.

Instructions for part 4 (on screen)

This part of the experiment is carried out as described on the instruction sheet. However, some details are different. Therefore, please read the following information carefully:

- At the beginning of a new round you and three randomly selected participants will form a group. For simplicity, you will always be presented as player 1, while your co-players will be presented as players 2, 3, and 4. However, the actual player behind a certain name (e.g. player 2) will change from round to round.

- Please note that unless you pick heads or tails, the round ends after turn 6.
- This part of the experiment consists of 7 rounds.
