# Supplementary material for: Experimental Evidence on Iterated Reasoning in Games
Source: PLoS One. 2015 Aug 27;10(8):e0136524. doi: 10.1371/journal.pone.0136524 (PMC4552469; doi:10.1371/journal.pone.0136524)
Supplement: S1 Appendix — (PDF) [file pone.0136524.s002.pdf]

S2 Appendix

In the appendix we provide the extended descriptions of the hit game and the dirty faces game, identify their solutions, and provide intuition why these solutions should obtain.

Hit game

A hit game is defined by a tuple (m, a, b) with $m, a, b \in \mathbb{N}\setminus\{0\}$ and $1 \leq a < b$. Two players alternately pick an integer from the interval $[a, b]$. These numbers are added up. The player who reaches m or surpasses it wins the game. Note, that each game (m, a, b) with any history and the current sum d is equivalent to a game (m', a, b) with $m' = m - d$. In other words, each current game can be reduced to a 'smaller' game in which the player having the move is the new starting player and the current sum d is set back to 0. Hence, it is sufficient to analyze only the first choice of the starting player for all possible games (m, a, b) in order to analyze all possible games and game histories.

We state that that the starting player is in a winning position if

$$m \in W := \bigcup_{k\in\mathbb{N}} \left[1 + k\left(a+b\right); b + k\left(a+b\right)\right]$$

and in a losing position if

$$m \in L := \bigcup_{k\in\mathbb{N}} \left[b + 1 + k\left(a+b\right); \left(k+1\right)\left(a+b\right)\right].$$

Note that winning and losing positions are mutually exclusive, i.e. $W \cap L = \emptyset$, and well defined over $\mathbb{N}$, i.e. $\forall m \in \mathbb{N}\setminus\{0\} : m \in W \vee m \in L$. Hence for each value of m the player is either in a winning or in a losing position.

In a subgame perfect equilibrium the player in a winning position can certainly achieve a victory by consistently forcing her opponent into a losing position, unless a direct win is possible. The player in the losing position is incapable of influencing this outcome. Our proof is three-folded: First, confirm that a direct win is only possible in a winning position with $k \doteq 0$. This is true because it is the only position from which the game can be won immediately by picking b (because of $m \leq b$). In any other position we have $m > b$ and a direct win is therefore impossible. Second, we prove that in a losing position the next

player will be in a winning position regardless of the number the current player is picking. To see this, let x be the number the player picks and m' be the by x reduced game for the next player. We can redefine m in a losing position as $m = b + 1 + k(a + b) + y$ with $k \in \mathbb{N}$ and $\mathbb{N} \ni y \in [0, a - 1]$. If we take into account that $(y - x) \in [-b; -1]$ we see that the reduced game

$$m' = m - x = b + 1 + k(a + b) + y - x \in [1 + k(a + b), b + k(a + b)] \subseteq W$$

must be a winning position.

Finally, we show that in any winning position with $k > 0$ the player can force the other player into a losing position by picking the number $max\{a, m\,mod\ (a + b)\}$. To see this, let m' be the reduced game for the next player. If we redefine m in a winning position as $m = 1 + k(a + b) + z$ with $k \in \mathbb{N}$ and $\mathbb{N} \ni z \in [0, b - 1]$ we have

$$\begin{aligned} m' &= m - max\{a, m\,mod\ (a + b)\} \\ &= 1 + k(a + b) + z - max\{a, (1 + k(a + b) + z)\ mod\ (a + b))\} \\ &= 1 + k(a + b) + z - max\{a, 1 + z)\}. \end{aligned}$$

In the case of $a \geq 1 + z \Leftrightarrow z < a$ we see that the reduced game

$$m' = 1 + k(a + b) + z - a = b + 1 + k'(a + b) + z \in L,\ k' \in \mathbb{N}$$

is a losing position. In the case of $a < 1 + z \Leftrightarrow z > a$ we have

$$m' = 1 + k(a + b) + z - (1 + z) = (k' + 1)(a + b) \in L,\ k' \in \mathbb{N},$$

which is also a losing position. □

Dirty Faces Game

In a dirty faces game each player $i \in N = \{1, 2, \ldots, n\}$ is assigned a type $\tau_i \in \{X, O\}$. Each player knows the types of all others but not her own. However, it is publicly announced that at least one player is an X-type. The game then proceeds in turns, with players privately choosing one of the three possible announcements 'I am an X-type' (X), 'I am an O-type' (O), or 'I don't know

my type' (U). When everyone has chosen an announcement, these are made public and a new turn begins. The game ends for a player by either announcing a certain type (X or O) or at the end of turn $T \geq n$. The incentives are designed in such a way that the players have an interest in logically deducing their own type and publicly announcing it as quickly as possible. In addition if a player isn´t capable of deducing her type yet she will announce U instead (see section 3.3 for details).

Following common practice in the experimental literature on the dirty faces game (Weber 2001; Bayer & Chan 2007), we abstain from a complete analysis of this game and instead focus on the underlying intuition in terms of interactive knowledge. Let $k \in \mathbb{N}$ with $k \leq n$ be the number of X-types in the game and $k_i \in \mathbb{N}$ the number of X-types a player i observes. Note that a player i observing k_i X-types knows that the true value of k must be either k_i (if $\tau_i = O$) or $k_i + 1$ (if $\tau_i = X$). Second, note the dual function of the public announcement: Besides providing each player with the private information that there is at least one X-type ($k \geq 1$) it makes this information common knowledge ($C\mathcal{K}(k \geq 1)$), i.e. everyone now knows this ($\mathcal{K}^1(k \geq 1)$) and everyone knows that everyone knows this ($\mathcal{K}^2(k \geq 1)$) and so on (until $\mathcal{K}^\infty(k \geq 1)$). We will show by induction that if all players are rational and this is common knowledge in the dirty faces game each player i is able to deduce her type for any $k_i \in \mathbb{N}$ with $0 \leq k_i < n$.

If $k_i = 0$ it is clear that i, observing only O-types (and learning this way $k \leq 1$), can immediately after the public announcement deduce that she has to be an X-type, which leads her to announce X in the first turn. In the case of $k_i = 1$ the only thing new i derives from the public announcement is $C\mathcal{K}(k \geq 1)$. Therefore, i is not able to deduce her type in turn 1 and will announce U. At the beginning of the new turn, however, i can see the announcement of the observed X-type j. If j has announced X in turn 1, he could do so only if $k_j = 0$ and if j knows that $k \geq 1$ (and i knows that j knows that because of $C\mathcal{K}(k \geq 1)$), hence j must be the only X-type in the game and $k = k_i \Leftrightarrow \tau_i = O$, therefore i announces O in turn 2. If, instead, j has announced U, i notices that j did not announce X in turn 1, hence this could only mean that $k_j > 0$ which implies that $k \geq 2 \Leftrightarrow \tau_i = X$. Note that we do not have to discuss the case that an X-type announces O, simply because this would contradict the rationality assumption. Also take into account that (at least for $k_i \leq 1$ it was the case that) a player i can infer her type exactly in turn $t = k_i + 1$.

Finally, let $d \in \mathbb{N}$ be the number for which we have shown that each player j observing $k_j \leq d$ can deduce his type at turn $t = k_j + 1$. We will show that

a player i observing $k_i = d+1$ can deduce her type in turn $t = k_i + 1$. In the case of $\tau_i = O$ the X-types, player i observes, observe only d other X-types, hence (using the induction basis) they can deduce their types in turn $d+1$ and will simultaneously announce X in the respective turn. Player i observing their announcement in turn $d+2$ knows thanks to $C\mathcal{K}\,(k \geq 1)$ on which basis they are acting and can therefore infer that $k = k_i \Leftrightarrow \tau_i = O$. In the case of $\tau_i = X$ the observed X-types observe $d+1$ other X-types, hence they will announce U in turn $d+1$. Player i, noticing in turn $d+2$ that the observed X-types did not announce X, and can therefore infer that $k > k_i \Leftrightarrow \tau_i = X$. □

In the equilibrium we observe that until turn $k-1$ all player announce U. At the beginning of each turn t it becomes common knowledge that $k \geq t$ (because otherwise the X-types would have had announced X already). In turn k all X-types learn that $k > k_i \Leftrightarrow \tau_i = X$ and simultaneously announce X, while the O-types continue in announcing U. In turn $k+1$ the O-types learn $k = k_i \Leftrightarrow \tau_i = O$ and simultaneously announce O.
